# Sputum microbiota and inflammatory subtypes in asthma, COPD, and its overlap

**DOI:** 10.1016/j.jacig.2023.100194

**Published:** 2023-11-21

**Authors:** Chie Morimoto, Hisako Matsumoto, Natsuko Nomura, Hironobu Sunadome, Tadao Nagasaki, Susumu Sato, Atsuyasu Sato, Tsuyoshi Oguma, Isao Ito, Mariko Kogo, Keisuke Tomii, Tomoko Tajiri, Kai Ohashi, Takamitsu Tsukahara, Toyohiro Hirai

**Affiliations:** a Department of Respiratory Medicine, Graduate School of Medicine, Kyoto University, Kyoto, Japan; b Department of Respiratory Medicine and Allergology, Faculty of Medicine, Kindai University, Osaka, Japan; c Department of Respiratory Care and Sleep Control Medicine, Kyoto University Graduate School of Medicine, Kyoto, Japan; d Department of Respiratory Medicine, Kobe City Medical Center General Hospital, Kobe, Japan; e Department of Respiratory Medicine, Japanese Red Cross Wakayama Medical Center, Wakayama, Japan; f Department of Respiratory Medicine, Allergy and Clinical Immunology, Graduate School of Medical Sciences, Nagoya City University, Nagoya, Japan; g Kyoto Institute of Nutrition & Pathology, Inc, Kyoto, Japan

**Keywords:** Asthma-COPD overlap, eosinophil-low, mixed granulocytic subtype, sputum microbiome

## Abstract

**Background:**

Airway microbiota in asthma-chronic obstructive pulmonary disease (COPD) overlap (ACO) remains unknown.

**Objective:**

This study with ACO-enriched population aimed to clarify airway microbiota in ACO and in mixed granulocytic inflammation, often detected in ACO and chronic airway diseases.

**Methods:**

This is an observational cross-sectional study. Patients with asthma with airflow limitation, ACO, and COPD were enrolled. Blood tests, pulmonary function, exhaled nitric oxide, and sputum tests were conducted. Sputum microbiota was evaluated using the 16S rRNA gene sequencing technique.

**Results:**

A total of 112 patients (13 asthma, 67 ACO, and 32 COPD) were examined. There were no significant differences in α-diversity among the 3 diseases. The relative abundances of phylum Bacteroidetes, class Bacteroidia, and genus *Porphyromonas* were associated with decreased eosinophilic inflammation, and were significantly lower in ACO than in COPD. In a comparison of sputum inflammatory subtypes, the proportion of *Haemophilus* was numerically highest in the mixed granulocytic subtype, followed by the neutrophilic subtype. Likewise, the proportion of *Haemophilus* was the highest in the intermediate-high (2%-8%) sputum eosinophil group and lowest in the severe (≥8%) eosinophil group. Clinically, *Haemophilus* proportion was associated with sputum symptoms. Finally, the proportion of *Streptococcus* was associated with higher blood eosinophil counts and most severe airflow limitation.

**Conclusions:**

Bacteroidia and *Porphyromonas* abundances in sputum are associated with the eosinophil-low phenotype, and ACO may be characterized by a decrease in these taxa. A mild elevation in sputum eosinophil does not preclude the presence of *Haemophilus*, which should be noted in the management of obstructive airway diseases.

There is increasing evidence that airway microbiota in chronic airway diseases, particularly chronic obstructive pulmonary disease (COPD), differs from that of healthy individuals, and is associated with disease pathophysiology and disease prognosis.^1^^,^^2^ Microbiota changes also occur in the lower airways of asthma, especially severe asthma,^3^ obese asthma,^4^ or asthma complicated by bronchiectasis.^5^^,^^6^ Another phenotype of airway disease that should be discussed for microbiota changes may be asthma and COPD overlap (ACO). Patients with ACO often experience more severe conditions than those with each disease alone and are at risk of bacterial infection. However, the information on microbiota on the lower airway of ACO is limited.^7^

Across the airway diseases, airway microbiota composition is often stated to relate to airway inflammatory subtypes. Neutrophilic inflammation has been repeatedly detected in the airways where the phylum Proteobacteria, which includes class γ Proteobacteria with a large number of pathogenic Gram-negative rods such as *Haemophilus* spp, is the predominant flora over the phylum Firmicutes, which includes Gram-positive cocci such as *Streptococcus* spp.^8^, ^9^, ^10^ In addition, steroid-naive patients with eosinophil-low asthma had more abundant genus *Bacteroides* in the lower airways than those with eosinophil-high asthma,^11^ and operational taxonomic units of *Bacteroides* sp. in the gut were negatively associated with blood eosinophil percentage in patients with COPD,^12^ suggesting that *Bacteroides* may be linked to eosinophil-low phenotype. Therefore, the neutrophil-high or eosinophil-low phenotype may have specific microbiota composition in the airways, but it remains unknown for the microbiome pattern in mixed granulocytic inflammatory subtype, where neutrophils and eosinophils coexist. Some patients with asthma with airflow obstruction, poor disease control,^13^, ^14^, ^15^ obesity,^16^ and chronic rhinosinusitis,^17^ or ACO^18^ possess mixed granulocytic airway inflammation. Hence, it is critical to comprehend mixed granulocytic airway inflammation in chronic airway diseases, but microbiota in mixed granulocytic airway inflammation remains unclear.^8^^,^^19^

The primary objective of this study was to identify airway microbiota in ACO and to determine the associations between airway microbiota, inflammatory subtypes, especially mixed granulocytic subtype, and clinical indices, simply concentrating on classes Bacteroidia, γ Proteobacteria, and Bacilli and genera *Bacteroides*, *Haemophilus*, and *Streptococcus* in ACO-enriched population. Genus *Porphyromonas* was another target of interest due to its involvement in periodontitis, a disease known to have an epidemiological relationship with COPD.

## Methods

### Subjects

This observational cross-sectional study enrolled patients with asthma with airflow limitation (FEV_1_/forced vital capacity < 70%), ACO, or COPD from January 2019 to February 2020 at Kyoto University Hospital and 2 collaborative institutions (Kobe City Medical Centre General Hospital and Japanese Red Cross Wakayama Medical Center) in Japan, when they were stable. The diagnosis was verified following the guideline for the management of ACO edited by the Japanese Respiratory Society (see Table E1 in this article’s Online Repository at www.jaci-global.org).^20^ The study protocol (R1705-1, UMIN000013020) was approved by the Ethics Committee of Kyoto University, and all participants provided written informed consent.

### Measurements

Blood tests, pulmonary function tests including the diffusing capacity for carbon monoxide, fractional exhaled nitric oxide (Feno), and sputum tests were carried out and the values were measured. Feno levels were determined during constant exhalation at a flow rate of 50 mL/s using a NIOX VERO (NOV; Aerocrine, Solna, Sweden) according to current guidelines of the American Thoracic Society.^21^ Measurements were conducted within 3 months of the date of consent acquisition. Details of other clinical data are described in this article’s Online Repository at www.jaci-global.org.

### Sputum microbiota analysis and cell differential count

Sputum, either induced or spontaneous, was obtained after gargling. Details of the induced sputum process have been described previously^22^ and definitions of sputum inflammatory subtypes are given in this article’s Online Repository at www.jaci-global.org. For microbiota analysis, bacterial DNA was extracted by using a commercial extraction kit (QuickGene DNA tissue kit; KURABO, Osaka, Japan) as previously described^23^ with some modifications. Details of the DNA extraction and its preparation,^23^ and microbiological analysis,^24^^,^^25^ are given in this article’s Online Repository at www.jaci-global.org. Microbiota with a relative abundance of 1% or more were assessed. The relative abundance of genera *Pseudomonas* and *Bacteroides* was less than 1%, but were evaluated because they were considered as important bacteria. In this study, we focused on classes Bacteroidia*,* γ Proteobacteria, and Bacilli and genera *Porphyromonas*, *Haemophilus*, and *Streptococcus.*

### Statistical analyses

Statistical analyses were performed using the JMP System, version 15 pro (SAS Institute Japan, Tokyo, Japan). Details are given in this article’s Online Repository at www.jaci-global.org. A *P* value of less than .05 was considered significant. Values are presented as mean ± SD.

## Results

In a total of 124 recruited patients, 112 patients were finally enrolled and assessed for sputum microbiota; 12 patients were excluded owing to FEV_1_/forced vital capacity greater than or equal to 70% at enrollment, missing pulmonary function test, or missing blood test (see Fig E1 in this article’s Online Repository at www.jaci-global.org). Of the 112 patients, 13 were categorized as suffering from asthma with airflow limitation, 67 as ACO, and 32 as COPD. The clinical characteristics of the 3 diseases are given in Table I.^26^ The degree of airflow limitation in patients with asthma was milder than that observed in patients with ACO (mean FEV_1_/FVC: 60.9% ± 9.3%, 55.4% ± 9.2%, and 53.1% ± 10.8% for asthma, ACO, and COPD, respectively).

**Table I:** Baseline characteristics of patients with asthma with airflow limitation, ACO, and COPD

Characteristic	All (N = 112)	Asthma (N = 13)	ACO (N = 67)	COPD (N = 32)	*P* value
Sex: male, n (%)	88 (79)	7 (54)	54 (81)∗	27 (84)∗	.06
Age (y)	72.0 ± 8.8	73.6 ± 7.7	70.6 ± 9.8	74.3 ± 6.4	.13
Body mass index (kg/m^2^)	23.4 ± 3.5	23.7 ± 3.7	23.5 ± 3.7	23.2 ± 3.1	1.0
Smoking: current/ex/never, n	13/70/29	0/2/11	10/43/14	3/25/4	<.0001
Pack-year	31.5 ± 32.5	0.95 ± 2.1	31.9 ± 31.8∗†	50.0 ± 30.2†	<.0001
ACT score (n = 104)	24 (9-25)	23.5 (19-25)	24 (9-25)	24 (12-25)	.57
CAT score (n = 104)	9 (0-36)	8 (0-28)	9 (0-27)	7 (0-36)	.20
ABCD assessment‡: A/B/C/D, n	83/8/13/8	11/1/0/1	43/6/12/6	29/1/1/1	.12
No. (%) of patients who had exacerbations in the previous year§	28 (25)	1 (8)	21 (31)	6 (19)	.12
FEV_1_/FVC (%)	55.4 ± 9.9	60.9 ± 9.3	55.4 ± 9.2∗	53.1 ± 10.8	.03
FEV_1_ (%predicted)	69.2 ± 18.5	81.6 ± 18.2	68.1 ± 16.8∗	66.4 ± 20.6	.04
%FEV_1_ (≥50%/50%∼30%/>30%), n	91/18/3	12/1/0	55/11/1	24/6/2	.51
%D_Lco_/VA (%) (n = 108)	92.2 ± 23.8	112.6 ± 15.1	90.9 ± 23.5∗	86.1 ± 23.4∗	.002
Sputum eosinophil (%) (n = 73)	4 (0-85)	4.3 (1.7-64)	5.4 (0-85)	2.1 (0-24)	.16
Sputum eosinophilia (%) (≥ 2%)	68.5	88.9	71.7	50.0	.03
Sputum neutrophil (%) (n = 73)	82 (14-99)	79 (28-95)	80 (14-99)	87 (55-99)	.13
Sputum neutrophilia (%) (≥ 60%)	75.3	55.6	71.7	94.4	.02
Exhaled nitric oxide (ppb) (n = 107)	46 ± 40	61 ± 41	54 ± 44†	23 ± 10∗	<.0001
Blood eosinophils (cells/μL)	338 ± 342	522 ± 606†	360 ± 316†	218 ± 179	.007
Blood neutrophils (cells/μL)	3913 ± 1141	4089 ± 1471	4080 ± 1110†	3492 ± 971	.04
Serum C-reactive protein (mg/dL)	0.2 ± 0.6	0.3 ± 0.9	0.2 ± 0.6	0.1 ± 0.2	.9
Serum total IgE (IU/mL) (n = 111)	190 (0-9822)	210 (0-110)	270 (9-9822)†	70 (8-880)	.004
Regular ICS use, n (%)	86 (77)	13 (100)	63 (94)	10 (31)∗	<.0001
ICS dose (μg/d)||	354 ± 322	479 ± 313	455 ± 308†	93 ± 179∗	<.0001
Regular OCS use, n (%)	11 (10)	2 (15)	9 (13)†	0 (0)∗	.09
Chronic rhinosinusitis, n (%)	26 (23)	2 (15)	19 (28)	5 (16)	.29
FSSG score ≥ 8, n (%)	26 (23)	3 (23)	19 (28)	4 (13)	.22

*ACT*, Asthma Control Test; *CAT*, COPD assessment test; *D*_*Lco*_*/VA*, diffusion capacity of carbon monoxide/alveolar volume; *FSSG*, frequency scale for the symptoms of gastroesophageal reflux; *mMRC*, modifed Medical Research Council dyspnea questionnaire.

*P* values in the last column present variance across 3 diseases. Data are presented as mean ± SD or median (range), otherwise stated specifically.

∗ *P* < .05 vs asthma.

† *P* < .05 vs COPD.

‡ According to Global Initiative for Chronic Obstructive Lung Disease, 2021 Report.^26^ Group A, low symptoms, ie, mMRC 0-1 or CAT < 10 and low exacerbation risk, ie, 0 or 1 moderate exacerbation, not leading to hospital admission; Group B, high symptoms, ie, mMRC ≥ 2 or CAT ≥ 10 and low exacerbation risk; Group C, low symptoms and high exacerbation risk, ie, ≥2 moderate exacerbations or ≥1 leading to hospitalization; Group D, high symptoms and high exacerbation risk.^26^

§ Cases of exacerbations that required antibiotics, systemic corticosteroids, emergency room visits, or hospitalization.

|| Equivalent to fluticasone propionate.

Induced sputum was obtained from 31 patients, whereas spontaneous sputum was obtained from 81 patients. Most patients with induced sputum were females and inhaled corticosteroid (ICS) users (see Table E2 in this article’s Online Repository at www.jaci-global.org). There were significant differences in the frequency of sputum eosinophilia (≥2%) and Feno level across asthma with airflow limitation, ACO, and COPD, with being lowest in COPD (mean sputum eosinophil: 12.0% ± 20.0%, 13.8% ± 20.1%, and 4.7% ± 6.1% for asthma, ACO, and COPD, respectively; mean Feno: 61 parts per billion [ppb] ± 41 ppb, 54 ppb ± 44 ppb, and 23 ppb ± 10 ppb, for asthma, ACO, and COPD, respectively), whereas the frequency of sputum neutrophilia was highest in COPD.

### Airway microbial community among patients with asthma with airflow limitation, ACO, and COPD

**Fig 1:**
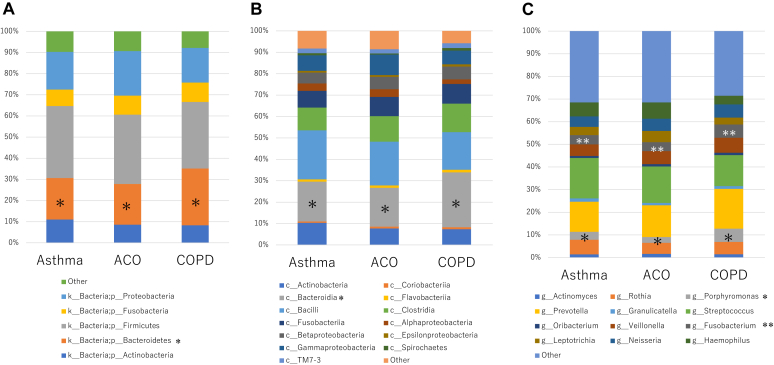
Sputum microbiota among patients with asthma with airflow limitation, ACO, and COPD. Relative abundances of bacteria at levels of (**A**) phylum, (**B**) class, and (**C**) genus. Bacteria for which relative abundance is 1% or more are presented. ∗The relative abundances of phylum Bacteroidetes,∗ class Bacteroidia,∗ and genera *Porphyromonas*∗ differed significantly across the 3 diseases (*P* < .05 by the Kruskal-Wallis test) with differences between COPD and ACO (*P* < .05 by the Steel-Dwass test for all). †The relative abundance of *Fusobacterium* differed significantly across the 3 diseases (*P* < .05 by the Kruskal-Wallis test) with differences between COPD and ACO (*P* < .05 by the Steel-Dwass test for all).

No significant differences were observed in Chao 1 and Shannon index, which express α-diversity among the 3 diseases (data not shown). β-Diversity analysis at the genus level varied significantly between patients with COPD and patients with ACO (*P* = .003), and between patients with COPD and patients with asthma (*P* = .016) (see Fig E2 in this article’s Online Repository at www.jaci-global.org). The relative abundances of phylum Bacteroidetes, class Bacteroidia, and genera *Porphyromonas* and *Fusobacterium* varied across the 3 diseases (*P* = .0003, *P* = .0005, *P* = .003, and *P* = .035, respectively), with significant differences between COPD and ACO (*P* < .05 for all) (Fig 1). Although spontaneous sputum samples demonstrated greater relative abundances of phylum Bacteroidetes, class Bacteroidia, and genus *Porphyromonas* as compared with induced sputum samples, the relative abundances of these bacteria in ACO were significantly lower than those in COPD even after adjusting for age, sex, pack-year, serum total IgE, daily doses of ICS, and type of sputum sampling (for *Porphyromonas*, see model 1 in Table E3 in this article’s Online Repository at www.jaci-global.org). Genus *Fusobacterium* was also significantly lower in ACO than in COPD (see model 2 in Table E3). The relative abundance of genus *Bacteroides* was less than 1% and was not different across the 3 diseases. No significant differences were observed in the relative abundances of other bacteria, including classes γ Proteobacteria and Bacilli and genera *Haemophilus* and *Streptococcus* across the 3 diseases.

### Class Bacteroidia abundance is associated with low sputum eosinophils

**Fig 2:**
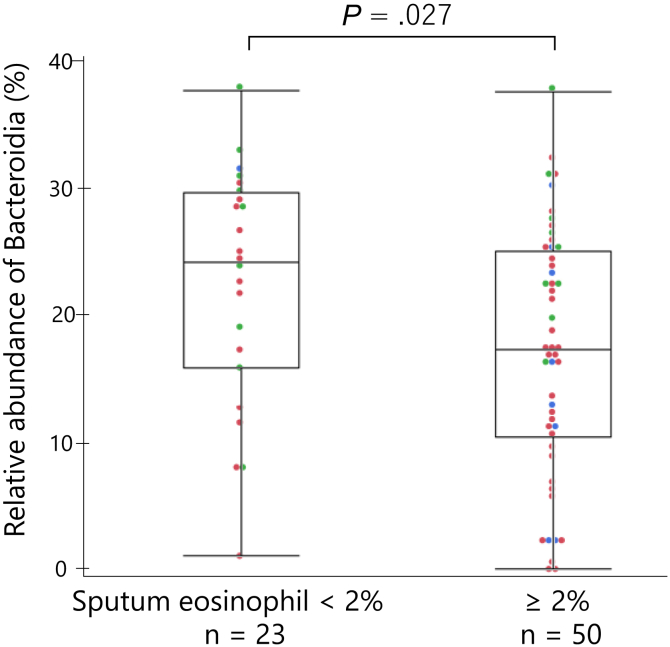
The relative abundances of class Bacteroidia of the eosinophil-low (sputum eosinophil < 2%) phenotype and the remaining (sputum eosinophil ≥ 2%). Boxes and bars indicate upper, lower, and median quartiles. Blue dots indicate patients with asthma; red dots, ACO; green dots, COPD. *P* = .027 by the Wilcoxon rank-sum test.

Among 112 patients, sputum differential cell counts were evaluated in 73 patients. Patients with sputum eosinophil less than 2% (n = 23) exhibited significantly higher relative abundance of phylum Bacteroidetes (*P* = .033) and class Bacteroidia than those with sputum eosinophil greater than or equal to 2% (n = 50) (*P* = .027) (Fig 2). The difference was marginally significant in phylum Bacteroidetes and remained significant in class Bacteroidia after adjustment for daily ICS doses and type of sputum sampling (see Table E4 in this article’s Online Repository at www.jaci-global.org). Likewise, there were weak negative associations between the relative abundance of Bacteroidia and sputum or blood eosinophil counts (*ρ* = −0.23, *P* = .040; *ρ* = −0.21, *P* = .032, respectively). The relative abundance of genus *Porphyromonas* was negatively associated with Feno (*ρ* = −0.22, *P* = .021). The relative abundance of genera *Fusobacterium* and *Bacteroides* was not associated with sputum or blood eosinophil counts and Feno (data not shown). The correlation coefficients between the relative abundance of bacteria and sputum eosinophil counts or Feno are presented in Table E5 (in the Online Repository available at www.jaci-global.org).

### Class γ Proteobacteria abundance is higher in neutrophilic and mixed granulocytic sputum subtypes

**Fig 3:**
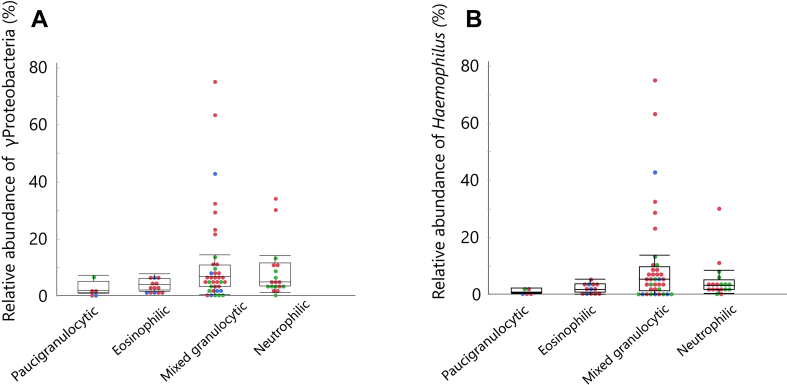
The relative abundances of (**A**) class γ Proteobacteria (*P* = .04 across the 4 subtypes by the Kruskal-Wallis test) and (**B**) genus *Haemophilus* (*P* = .02 across the 4 subtypes by the Kruskal-Wallis test) according to the sputum inflammatory subtypes: paucigranulocytic (n = 5), eosinophilic (n = 13), mixed granulocytic (n = 37), and neutrophilic (n = 18). Boxes and bars indicate upper, lower, and median quartiles. Blue dots indicate patients with asthma; red dots, ACO; green dots, COPD.

As expected, sputum neutrophil count was weakly but significantly correlated with the relative abundances of the class γ Proteobacteria (*ρ* = 0.35, *P* = .002) and genus *Haemophilus* (*ρ* = 0.33, *P* = .005). Furthermore, sputum neutrophilia (neutrophil ≥ 60%) was significantly associated with a higher proportion of class γ Proteobacteria and genus *Haemophilus* after adjustment with confounding factors (data not shown). Meanwhile, when stratified by sputum inflammatory subtypes, that is, paucigranulocytic (n = 5), eosinophilic (n = 13), mixed granulocytic (n = 37), and neutrophilic (n = 18) subtypes, the relative abundances of γ Proteobacteria and *Haemophilus* differed significantly across the 4 subtypes (Table II) and were numerically highest in the mixed granulocytic subtype, followed by the neutrophilic subtype (Fig 3). This also holds when the cutoff value of sputum eosinophil was shifted to 3% (*P* = .028 across the 4 inflammatory subtypes). The relative abundances of bacterium at the phylum, class, and genera levels across the 4 inflammatory subtypes are shown in Fig E3 (in the Online Repository available at www.jaci-global.org).

**Table II:** Baseline characteristics stratified by sputum inflammatory subtypes

Characteristic	Paucigranulocytic (N = 5)	Eosinophilic (N = 13)	Mixed granulocytic (N = 37)	Neutrophilic (N = 18)	*P* value
Sex: male, n (%)	4 (80)	11 (85)	28 (76)	16 (89)	.68
Age (y)	70 ± 6	71 ± 11	70 ± 9	71 ± 10	.84
Body mass index (kg/m^2^)	26.5 ± 5.0	23.6 ± 4.0	23.7 ± 2.9	24.3 ± 2.9	.42
Smoking: current/ex/never	2/2/1	0/10/3	8/18/11	1/14/3	.14
Asthma/ACO/COPD, n	1/3/1	3/10/0	5/23/9	0/10/8	.11
No. (%) of patients who had exacerbations in the previous year∗	2 (40)	4 (31)	12 (32)	1 (6)	.15
FEV_1_/FVC (%)	57.1 ± 6.6	58.2 ± 8.3	55.4 ± 8.7	55.7 ± 11.6	.78
FEV_1_ (%predicted)	68.6 ± 15.2	69.7 ± 5.4	71.5 ± 3.2	70.4 ± 4.6	.97
Sputum eosinophil (%)	1.5 (0-1.7)	36 (2-85)	7 (2-27)	0.9 (0-1.5)	—
Sputum eosinophil<2%/≥2% to <8%/≥8%	5/0/0	0/3/10	0/23/14	18/0/0	<.0001
Sputum neutrophil (%)	40 (29-56)	39 (14-59)	85 (61-96)	93 (69-99)	—
Exhaled nitric oxide (ppb)	24 ± 15	78 ± 65†‡	53 ± 37†	27 ± 17	.0003
Blood eosinophils (cells/μL)	169 ± 105	440 ± 223†	438 ± 508†	194 ± 183	.006
Blood neutrophils (cells/μL)	4248 ± 1666	3529 ± 492	4037 ± 1197	4192 ± 1341	.33
Serum C-reactive protein (mg/dL)	0.52 ± 1.1	0.11 ± 0.11	0.23 ± 0.60	0.15 ± 0.23	.63
Serum total IgE (IU/mL)	470 (8-530)	560 (34-1785)	170 (0-8600)	74 (9-9822)	.43
Relative abundance (%) of Class Bacteroidia	22.7 ± 9.0	14.2 ± 8.5	18.2 ± 9.8	22.4 ± 9.6	.09
Class γ Proteobacteria	2.5 ± 2.6	3.9 ± 2.2	12.2 ± 17.0	8.6 ± 9.5	.042
Class Bacilli	17.7 ± 4.8	20.7 ± 10.9	20.2 ± 12.1	16.5 ± 8.1	.51
Genus *Porphyromonas*	3.5 ± 2.3	1.6 ± 1.5	3.5 ± 3.9	4.0 ± 4.0	.24
Genus *Haemophilus*	1.3 ± 0.9	2.3 ± 1.7	10.9 ± 17.2	5.2 ± 6.9	.020
Genus *Streptococcus*	14.1 ± 3.7	16.2 ± 7.4	16.4 ± 10.6	13.7 ± 7.9	.59
ICS dose (μg/d)§	285 ± 295	453 ± 218	435 ± 344	313 ± 364	.28

*P* values in the last column present variance across 4 inflammatory subtypes.

Values are mean ± SD or median (range).

∗ Cases of exacerbations that required antibiotics, systemic corticosteroids, emergency room visits, or hospitalization.

† *P* < .05 vs the neutrophilic subtype.

‡ *P* < .05 vs the paucigranulocytic subtype.

§ Equivalent to fluticasone propionate.

**Fig 4:**
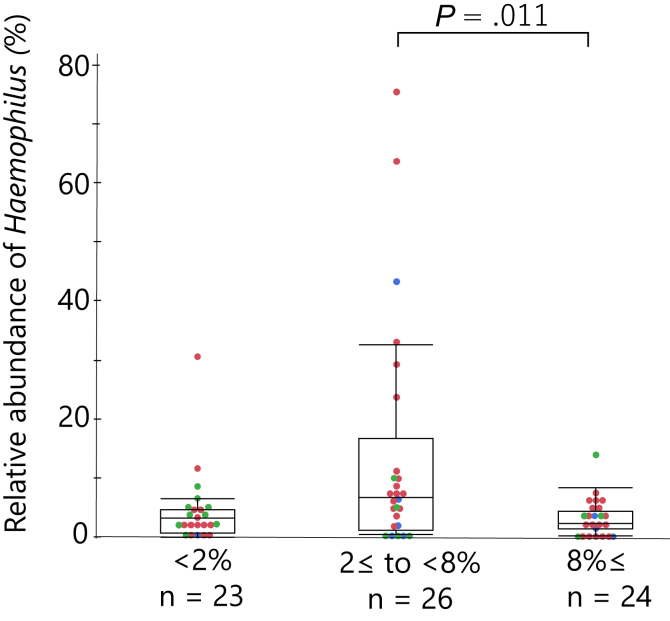
The relative abundances of genus *Haemophilus* based on sputum eosinophil levels (<2%, ≥2% to <8%, and ≥8%; *P* = .009 across the 3 groups by the Kruskal-Wallis test. *P* = .011, sputum eosinophil ≥2% to <8% group vs ≥8% group by the Steel-Dwass test). Boxes and bars indicate upper, lower, and median quartiles. Blue dots indicate patients with asthma; red dots, ACO; green dots, COPD.

In addition, when patients were stratified into 3 groups on the basis of sputum eosinophil counts alone, the relative abundance of *Haemophilus* was the lowest in patients with high (≥8%) level of sputum eosinophils, whereas it was the richest in patients with modestly elevated (from 2% to less than 8%) eosinophils (*P* = .011 vs high eosinophil group) (Fig 4), which remained significant after adjustment with the 3 diseases (see Table E6 in this article’s Online Repository at www.jaci-global.org).

### Genus *Streptococcus* abundance is associated with severe airflow limitation

**Fig 5:**
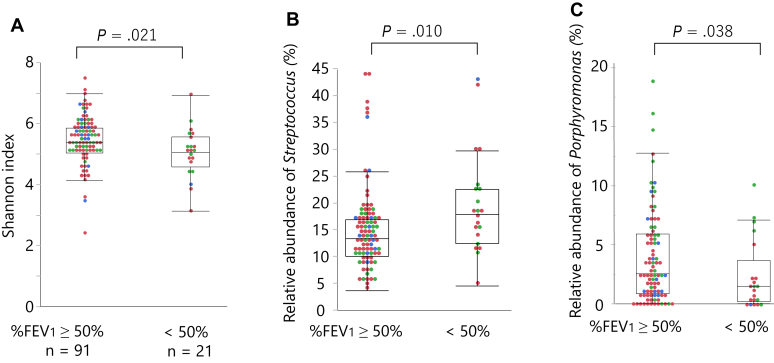
Sputum microbiota according to the degree of %FEV_1_. (**A**) Shannon index, and the relative abundance of genera (**B**) *Streptococcus* and (**C**) *Porphyromonas*. Two group comparisons were made using the Wilcoxon rank-sum test. Boxes and bars indicate upper, lower, and median quartiles. Blue dots indicate patients with asthma; red dots, ACO; green dots, COPD.

**Fig 6:**
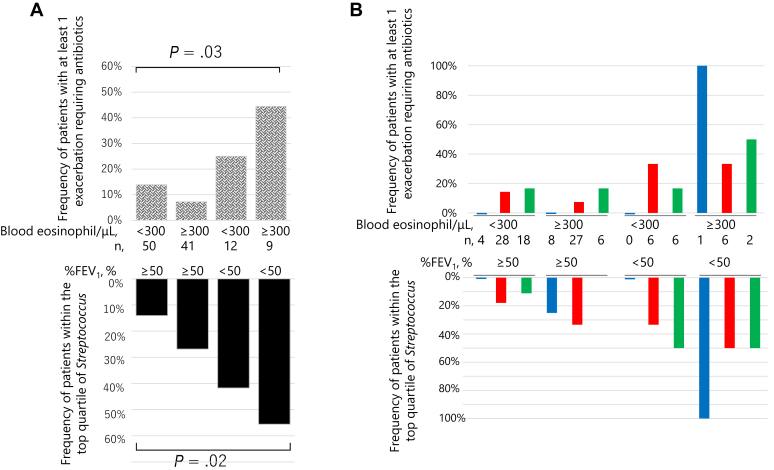
**A,** Frequencies of patients with at least 1 exacerbation necessitating antibiotics in the previous year, stratified by the presence or absence of blood eosinophilia and severe airflow limitation (*upper panel*) (*P* = .03 across the 4 groups, by the Kruskal-Wallis test) and those within the top quartile of *Streptococcus* (*lower panel*) (*P* = .02 across the 4 groups). **B,** Blue bars indicate patients with asthma; red bars, ACO; green bars, COPD.

Next, the 112 patients were divided into 2 groups on the basis of %FEV_1_: patients with %FEV_1_ greater than or equal to 50% (n = 91) and patients with %FEV_1_ less than 50% (n = 21). Patients with %FEV_1_ less than 50% had significantly higher frequencies of exacerbation, which necessitated antibiotics, systemic corticosteroids, or hospital admission in the previous year (see Table E7 in this article’s Online Repository at www.jaci-global.org). The relative abundances of bacterium at the phylum, class, and genera levels of the 2 groups are shown in Fig E4 (in the Online Repository available at www.jaci-global.org). Patients with %FEV_1_ less than 50% exhibited significantly lower Shannon index (Fig 5, *A*) and a significantly higher proportion of the phylum Firmicutes (*P* = .004), class Bacilli (*P* = .012), and genus *Streptococcus* (*P* = .010) (Fig 5, *B*) than those with %FEV_1_ greater than or equal to 50%, while lower in *Porphyromonas* (*P* = .038) (Fig 5, *C*) and *Haemophilus* (*P* = .046). The association of genera *Streptococcus* and *Porphyromonas* with %FEV_1_ less than 50% remained significant after adjustment with the 3 diseases (see Table E8 in this article’s Online Repository at www.jaci-global.org). Patients within the top quartile of genus *Streptococcus* relative abundance had a significantly higher frequency of exacerbations requiring antibiotics in the previous year than the remaining (0.61 ± 1.29 vs 0.12 ± 0.42, *P* = .003) and comprised more patients who had at least 1 exacerbation requiring antibiotics (32% vs 10%, *P* = .01) (see Fig E5 in this article’s Online Repository at www.jaci-global.org). Nevertheless, the significance disappeared when adjusted for severe airflow limitation (%FEV_1_ < 50%) (data not shown). The relative abundance of genus *Streptococcus* was not associated with sputum eosinophil levels or sputum inflammatory subtypes, but with blood eosinophil counts (*ρ* = 0.26, *P* = .006). Frequencies of patients with at least 1 exacerbation requiring antibiotics and those within the top quartile of *Streptococcus*, stratified by the presence or absence of blood eosinophilia (≥300 cells/μL) and severe airflow limitation, are shown in Fig 6. The correlation coefficients between the relative abundance of bacteria and %FEV_1_ or frequency of exacerbations requiring antibiotics in the previous year are presented in Table E5.

### Genus *Haemophilus* abundance is associated with sputum symptoms

Sputum microbiota exhibited no significant associations with asthma control test scores, comorbidities, or the regular use of macrolide. Patients with sputum symptom that was expressed by 3 or more scores of sputum item of COPD assessment test questionnaire (n = 24) demonstrated significantly higher relative abundances of classes γ Proteobacteria (*P* = .006) and Bacilli (*P* = .025), and genera *Haemophilus* (*P* = .037) and *Streptococcus* (*P* = .043), than the remaining (n = 80). Other clinical indices associated with sputum symptoms were higher blood and sputum eosinophil counts, Feno, and frequency scale for the symptoms of gastroesophageal reflux, and lower %FEV_1_ (all *P* < .05). The genus *Haemophilus* and sputum eosinophilia were independently associated with sputum symptoms in the multivariable analysis (see Table E9 in this article’s Online Repository at www.jaci-global.org). Finally, the relative abundance of the genus *Pseudomonas* was significantly associated with ICS dose in patients with regular oral corticosteroids (OCSs) (*ρ* = 0.78, *P* = .005, n = 11) (see Fig E6 in this article’s Online Repository at www.jaci-global.org). This association was not observed in patients treated with ICS alone.

## Discussion

The current investigation of chronic obstructive airway diseases enriched with patients with ACO newly showed that the relative abundances of class Bacteroidia and genus *Porphyromonas* were inversely correlated with sputum and blood eosinophil counts or Feno levels, and were significantly lower in ACO than in COPD. Moreover, the mixed granulocytic subtype, characterized by sputum neutrophilia and modest elevation of eosinophil, was associated with higher relative abundance of the class γ Proteobacteria and genus *Haemophilus*. The proportion of *Streptococcus* was associated with higher blood eosinophil counts and severe airflow limitation. Finally, *Haemophilus* proportion and sputum eosinophil counts were both independently associated with sputum symptoms.

This study newly demonstrated that the relative abundances of phylum Bacteroidetes, class Bacteroidia, and genus *Porphyromonas* were significantly lower in ACO than in COPD. In other words, these taxa were the highest in COPD among the 3 diseases. When considering that most patients in this study did not exhibit severe COPD, abundant Bacteroidetes in COPD is in line with previous findings that the relative abundances of phyla Bacteroidetes over Proteobacteria were significantly higher in moderate COPD than in severe COPD.^27^ More importantly, the abundance of the phylum Bacteroidetes and class Bacteroidia was associated with less eosinophilic/type-2 inflammation, whereas genus *Bacteroides* was not associated with eosinophil-low phenotype, possibly due to its low abundance in sputum. The mechanisms underlying the association between eosinophil-low and more abundant phylum Bacteroidetes or genera *Porphyromonas* and *Bacteroides* remain unknown, but butyrate, one of the short-chain fatty acids produced by *Porphyromonas* and *Bacteroides*, may play a role.^28^ There is growing evidence on the involvement of the bacterial metabolite butyrate in the suppression of type-2 or eosinophilic inflammation.^29^^,^^30^ Previous research including ours displays that butyrate induces eosinophil apoptosis^29^ and suppresses the proliferation of type-2 innate lymphoid cells^30^; β-hydroxybutyric acid, a structural analog of butyrate, is negatively associated with blood eosinophil counts.^31^ Although because these bacteria are obligate anaerobes, additional studies are required to determine whether they generate butyrate in the lower airways; increased phylum Bacteroidetes or class Bacteroidia may determine the eosinophil-low phenotype and decrease the possibility of ACO. Finally, it is important to note that the current findings were for stable conditions. A recent study of the sputum microbiome of ACO^7^ has revealed a significant decrease in the taxonomic richness and an increase in taxonomic evenness during exacerbation compared with the stable state, with a significant increase in *Fusobacteria* and a marginal increase in *Bacteroidetes* during the exacerbation phase. Further studies are necessary to determine the association of this increase with inflammatory phenotype changes.

Previous studies on sputum microbiome in asthma and COPD^9^^,^^32^ or in severe asthma^8^^,^^10^^,^^33^ showed an association between neutrophil-high phenotype and γ-proteobacteria or *Haemophilus*, and studies during exacerbations^27^ and at stable state^9^ identified a cluster mainly containing patients with COPD and some with asthma, which was characterized by an increased ratio of γ proteobacteria to *Firmicutes* and high IL1-β and TNF-α levels. Consistent with previous studies, relative abundances of γ Proteobacteria and *Haemophilus* were associated with airway neutrophilia in this study. We newly indicated that the relative abundances of γ Proteobacteria and *Haemophilus* were the highest in patients with the mixed granulocytic subtype and with eosinophils from 2% to less than 8%, implying that the modest eosinophilia coexisting with neutrophilia should be interpreted as equivalent to a neutrophilic group in regard to the presence of γ Proteobacteria and *Haemophilus*. A study in asthma revealed a high number of operational taxonomic units in sputum in the mixed granulocytic group, similar to the neutrophilic group,^19^ but the study revealed no specific bacterial pattern within the group. When considering that LPS-stimulated neutrophils increase chemotaxis of eosinophils,^34^ the coexistence of γ Proteobacteria and increased neutrophils may accumulate eosinophils in the lower airways and cause mixed granulocytic inflammation, but further studies are needed to establish a causal relationship.

As in severe eosinophilic asthma, where mycobiota or virus infection may be crucial,^35^ the burden of pathogenic bacteria, especially genus *Haemophilus*, appears to be smaller in severe eosinophilic inflammation than in neutrophilic or mixed granulocytic inflammation. Indeed, the proportion of phylum Proteobacteria or genus *Haemophilus* declines in patients with severe asthma with sputum eosinophilia^8^ or those with high Feno (≥50 ppb).^33^ However, eosinophilic inflammation may have an affinity for the genus *Streptococcus*, according to its significant association with blood eosinophilia. These are in line with previous studies on severe asthma in which sputum or blood eosinophil percentage was negatively associated with the relative abundance of the genus *Haemophilus*, and positively with the genus *Streptococcus*.^8^ Blood eosinophil counts were positively associated with *Streptococcus* and negatively with *Haemophilus* proportions in COPD.^2^ In another study, operational taxonomic units of *Streptococcus* in sputum were most strongly associated with the relatively recent onset of severe asthma with eosinophilia and a history of rhinosinusitis.^36^

Notably, in this ACO-enriched population, genus *Streptococcus* was associated with more severe airflow limitation and with exacerbations requiring antibiotics. The latter association was consistent with a previous study of COPD^2^ but disappeared after adjustment for severe airflow limitation (%FEV_1_ < 50%) in this study. The mechanisms of the association between *Streptococcus* and severe airflow limitation are unknown, but airflow limitation is previously portrayed to be associated with lower α-diversity in severe asthma^8^ and the greater abundance of pathogenic bacteria in COPD^37^^,^^38^ and both *Haemophilus*^39^ and *Streptococcus*^40^ are noted to be associated with severe airflow limitation in patients with COPD. ACO-enriched population with a relatively small number of subjects with severe COPD in this study may explain the association of severe airflow limitation with *Streptococcus* but not *Haemophilus*. Further studies using phenotypic analysis are required to generalize the link between microbiota composition and severe airflow limitation.

Sputum symptom is one of the important symptoms in the chronic obstructive airway diseases, and the importance of using the COPD assessment test to detect chronic mucus hypersecretion has been shown recently.^41^^,^^42^ We newly demonstrated, using the sputum item of the COPD assessment test, that sputum symptom was associated with higher relative abundances of genera *Streptococcus* and *Haemophilus* and airway eosinophilia, with the latter 2 remaining significant in the multivariable analysis. Lastly, the ICS dose was positively associated with the relative abundance of *Pseudomonas* in patients with regular OCS use in this study, which is consistent with a previous study reporting that patients with asthma treated with ICS and OCS demonstrated a greater abundance of *Pseudomonas* in endobronchial brushings than did steroid-naive patients.^43^ Similarly, previous reports showed that high-dose ICS was associated with reduced bacterial diversity^8^ and higher abundance of Proteobacteria^44^ in patients with asthma, implying the potentially reduced local immunity of the respiratory tract, specifically in patients using high doses of ICS and OCS.

As limitation, the sputum microbiota was evaluated only once in this cross-sectional observational study in a racially and ethnically homogeneous population. This might preclude the generalization of our findings to other regions. However, the microbiota of the lower respiratory tract seems to alter modestly over time, especially in patients with asthma^45^ and mild to moderate COPD.^27^ In addition, given that most of the bacteria analyzed in our study were previously reported ones, the results could be extrapolated to other regions as well. Next, sputum microbiota may not accurately show the lower airway microbiota, but it is extensively used in the study of airway diseases,^38^ using either induced sputum^3^ or spontaneous sputum^37^ or both,^7^^,^^9^^,^^33^ and can be a marker that offers insight into their pathophysiology. Indeed, an increased proportion of genera *Porphyromonas* and *Fusobacterium*, which are the prominent bacteria involved in periodontitis, in COPD may reflect the high prevalence of periodontitis in COPD in large epidemiological studies.^46^^,^^47^ Particularly, *Porphyromonas* is a representative periodontal pathogen that constitutes the “Red complex” and yields a large amount of butyrate in the progression of periodontal disease.^28^ Both spontaneous and induced sputum samples were used in this study, but the lower relative abundances of class Bacteroidia and genus *Porphyromonas* in ACO as compared with COPD remained significant when the analysis was confined to spontaneous sputum only (data not shown). Third, this observational study included a relatively small number of patients with asthma, because patients with airflow limitation were recruited to minimize the influence of differences in microbiota caused by the presence or absence of airflow limitation.^37^^,^^38^ In addition, some patients who had been managed as asthma demonstrated low diffusing capacity for carbon monoxide and were categorized into the ACO group on the basis of the the Japanese Respiratory Society guideline^20^ in this study, which may not hinder the analysis in this study focusing on ACO.

**Clinical implications**

Patients with ACO, often accompanied by mixed granulocytic airway inflammation, showed decreased *Porphyromonas*, which is associated with the eosinophil-low phenotype, and showed similar abundance of *Haemophilus*, when compared with COPD.

### Conclusions

The microbiota composition characteristic of ACO in comparison with COPD may be reduced Bacteroidetes, Bacteroidia, and *Porphyromonas*, which are associated with the eosinophil-low phenotype. In addition, mixed granulocytic inflammation or a mild elevation in sputum eosinophil does not preclude the presence of *Haemophilus* and need attention in obstructive airway disease management.

## Disclosure statement

This study was funded by the 10.13039/100019085Japanese Respiratory Foundation and the 10.13039/501100001691Japan Society for the Promotion of Science (grant nos. 19K08649 and 22K08271).

Disclosure of potential conflict of interest: The authors declare that they have no relevant conflicts of interest.
