# Supplementary material for: Sputum microbiota and inflammatory subtypes in asthma, COPD, and its overlap
Source: J Allergy Clin Immunol Glob. 2023 Nov 21;3(1):100194. doi: 10.1016/j.jacig.2023.100194 (PMC10753087; doi:10.1016/j.jacig.2023.100194)
Supplement: Supplementary data [file mmc1.pdf]

Clean version

Supplementary material

Sputum microbiota and inflammatory subtypes in asthma, COPD, and its overlap

Chie Morimoto, Hisako Matsumoto, Natsuko Nomura, Hironobu Sunadome, Tadao Nagasaki, Susumu Sato, Atsuyasu Sato, Tsuyoshi Oguma, Isao Ito, Mariko Kogo, Keisuke Tomii, Tomoko Tajiri, Kai Ohashi, Takamitsu Tsukahara, Toyohiro Hirai

Methods

Measurements

Clinical data described below were also assessed: asthma control test, COPD assessment test, and frequency scale for the symptoms of gastroesophageal reflux[1], comorbidities, exacerbation that necessitated antibiotics, systemic corticosteroids or hospital admission during the past 12 months, inhaled corticosteroid dose, and the regular use of macrolide and oral corticosteroid.

Sputum microbiota analysis and cell differential count

For microbiota analysis, bacterial DNA was extracted by commercial extraction kit (QuickGene DNA tissue kit; KURABO, Osaka Japan) as previously described[2] with some modifications. Briefly, 100 μL of mixture equivalent volume of sputum and Sputasol® (Thermo Fisher, UK) was obtained in a sterilized conical tube containing beads, centrifuged at 15,000g×5 min, at 4°C, and then the supernatant was removed. Slurry within the bacteria was suspended with 250 μL of a tissue lysis buffer contained in the kit. The beads, 10 of 1.0 φ zirconia beads (Toray Industries Inc., Tokyo, Japan) and 15 mg of 0.1φ glass beads (As One, Osaka, Japan) per specimen were applied to the tubes to crush bacterial cell walls efficiently. The suspensions were smashed twice at 3,000 rpm for 2 min by the Micro Smash MS-100 (TOMY, Tokyo, Japan). Twenty-five μL of proteinase K contained in the kit was added to suspensions and incubated at 55°C for 2 h in a dry bath. After centrifugation (1,5000g×10 min, 25°C), 200 μL of the supernatant for DNA extraction were transferred to a new microtube. One hundred and eighty microliters of the lysis buffer contained in the kit was applied to the supernatant. After incubation at 70°C for 10 min, 240 μL of ethanol (99% v/v; SIGMA-Aldrich Japan, Tokyo, Japan) were

added. The bacterial genomic DNA in the suspension was purified using the QuickGene 810 system (KURABO). DNA extracts was harvested from the purified column by extract buffer contained in the kit, and was stored at −20°C until analysis. Preparation of the library was carried out following “16S Metagenomic Sequencing Library Preparation” provided by Illumina Inc (SanDiego, CA, USA). The V3-4 region of 16S rRNA genes in each sample was amplified. Electrophoresis was carried out on the amplicon to validate that the specimen was polymerase chain reaction (PCR)-amplified, and then purified by NucleoFast 96 (Takara Bio Inc, Shiga, Japan). The total volume of the second PCR products was set to 40 μL, and the PCR product was purified using SequalPrep Normalization Plate Kit (Life Technologies, Tokyo, Japan). Each of the normalized amplicons was then evenly pooled and concentrated using AMPure XP beads (Beckman Coulter, Tokyo, Japan). Final DNA concentrations were verified by KAPA Library Quantification Kits (NIPPON Genetics, Tokyo, Japan). A reagent called PhiX Control v3 (Illumina) was added immediately before loading into the sequencer, which worked as a positive control. The sequencing reads were processed by using Miseq and QIIME1.9.1 pipeline, as described previously [3,4]. For the taxonomy assignment, Greengenes 13.8 was used. α-diversity was assessed by Chao 1 and Shannon index, and β-diversity was assessed by PERmutational Multivariate Analysis of Variance of weighted UniFrac distances.

A sputum differential cell count was also obtained from patients enrolled at the Kyoto University Hospital. Sputum inflammatory subtypes were determined as follows: eosinophilic subtype, when sputum eosinophils ≥ 2% [5] [6] and neutrophils < 60%; neutrophilic subtype, when sputum eosinophils < 2% and neutrophils ≥ 60%; mixed granulocytic subtype, when eosinophils ≥ 2% and neutrophils ≥ 60%; and pauci-

granulocytic subtype, when eosinophils < 2% and neutrophils < 60%.

Statistical analyses

As no previous studies carried out microbiome analysis in asthma and COPD (ACO)-enriched population, the sample size of this study was determined based on a previous study evaluating the microbiome profile of severe asthma and moderate to severe COPD during exacerbations[7]. Two or more groups were compared using the χ^2 test, Fisher's exact test, Wilcoxon rank-sum test, and Kruskal-Wallis test, where appropriate. Multiple comparison tests were conducted using Steel-Dwass test. Cochran-Armitage test for trend was used to assess the association between the frequency of sputum eosinophilia or neutrophilia and three diseases. Relationships between variables were assessed using Spearman's correlation coefficients. Logistic multivariable analysis was performed to determine variables associated with the diagnosis of ACO and other outcomes. In the logistic multivariable analysis, clinically important variables that showed significant differences in the univariable analysis were used as covariates, in addition to age and sex. The number of variables were determined to meet $1/10^{th}$ of the population of interest.

References

1. Morimoto C, Matsumoto H, Nagasaki T, Kanemitsu Y, Ishiyama Y, Sunadome H, et al. Gastroesophageal reflux disease is a risk factor for sputum production in the general population: the Nagahama study. Respir Res 2021; 22:6.
2. Tsukahara T, Inoue R, Nakayama K, Inatomi T. Inclusion of Bacillus amyloliquefaciens strain TOA5001 in the diet of broilers suppresses the symptoms of coccidiosis by modulating intestinal microbiota. Anim Sci J 2018; 89:679-87.
3. Inoue R, Sakaue Y, Sawai C, Sawai T, Ozeki M, Romero-Perez GA, et al. A preliminary investigation on the relationship between gut microbiota and gene expressions in peripheral mononuclear cells of infants with autism spectrum disorders. Biosci Biotechnol Biochem 2016; 80:2450-8.
4. Inoue R, Sawai T, Sawai C, Nakatani M, Romero-Perez GA, Ozeki M, et al. A preliminary study of gut dysbiosis in children with food allergy. Biosci Biotechnol Biochem 2017; 81:2396-9.
5. Hastie AT, Mauger DT, Denlinger LC, Coverstone A, Castro M, Erzurum S, et al. Mixed Sputum Granulocyte Longitudinal Impact on Lung Function in the Severe Asthma Research Program. Am J Respir Crit Care Med 2021; 203:882-92.
6. Abdo M, Pedersen F, Kirsten AM, Veith V, Biller H, Trinkmann F, et al. Longitudinal Impact of Sputum Inflammatory Phenotypes on Small Airway Dysfunction and Disease Outcomes in Asthma. J Allergy Clin Immunol Pract 2022; 10:1545-53 e2.
7. Ghebre MA, Pang PH, Diver S, Desai D, Bafadhel M, Haldar K, et al. Biological exacerbation clusters demonstrate asthma and chronic obstructive pulmonary disease overlap with distinct mediator and microbiome profiles. J Allergy Clin Immunol 2018; 141:2027-36 e12.
8. Hashimoto S, Sorimachi R, Jinnai T, Ichinose M. Asthma and Chronic Obstructive Pulmonary Disease Overlap According to the Japanese Respiratory Society Diagnostic Criteria: The Prospective, Observational ACO Japan Cohort Study. Adv Ther 2021; 38:1168-84.

Table E1. Japanese Respiratory Society diagnostic criteria for asthma and COPD overlap[8]

<table>
<tr><td colspan="2">Basic criteria</td></tr>
<tr><td colspan="2">Age ≥ 40 years and chronic airflow obstruction: post-bronchodilator $FEV_1/FVC < 70\%$</td></tr>
<tr><td>[Characteristics of COPD]
One item from 1, 2, and 3</td><td>[Characteristics of asthma]
Two items from 1, 2, and 3, or
One item from 1, 2, and 3 and at least two items from 4</td></tr>
<tr><td>1. Smoking history (10 pack-years or more) or career involving significant air pollution or biomass exposure
2. Presence of low attenuation areas on chest CT demonstrating emphysematous changes
3. Impaired pulmonary diffusing capacity ($\%D_{LCO} < 80\%$ or $\%D_{LCO}/V_A < 80\%$)</td><td>1. Variable (diurnally, daily, and seasonally) or paroxysmal respiratory symptoms (cough, sputum, and dyspnea)
2. History of asthma before age 40 years
3. FeNO > 35 ppb
4. (1) Concomitant perennial allergic rhinitis
(2) Airway reversibility (change in $FEV_1 > 12\%$ and > 200 mL)
(3) Peripheral blood eosinophils > 5% or > 300/μL
(4) High IgE level (total IgE, or IgE specific to perennial inhalant antigens)</td></tr>
<tr><td colspan="2">1. To be diagnosed as ACO, one item of the characteristics of COPD plus two items from 1, 2, and 3 or one item from 1, 2, and 3 and at least two items from criterion 4 of the characteristics of asthma are needed
2. If the characteristics of COPD alone are present, it is diagnosed as COPD, and if the characteristics of asthma alone are present, it is diagnosed as asthma (with remodeling)
3. If the characteristics of asthma cannot be confirmed when diagnosing ACO, it is important to monitor for the presence of the characteristics of asthma over time
4. Perennial inhalant antigens include house dust, mites, molds, scales from animals, and feathers, and seasonal inhalant antigens include pollen from trees, plants, and weeds
Note 1. Diseases of differential diagnosis (diffuse panbronchiolitis, congenital sinobronchial syndrome, obstructive panbronchiolitis, bronchiectasis, pulmonary tuberculosis, pneumoconiosis, lymphangioleiomyomatosis, congestive heart failure, interstitial lung disease, and lung cancer) should be ruled out by standard chest x-rays, etc.
Note 2. Respiratory symptoms such as cough, sputum, and dyspnea are variable (diurnally, daily, and seasonally) or paroxysmal in asthma and chronic and continuous in COPD</td></tr>
</table>

COPD chronic obstructive pulmonary disease, *CT* computed tomography, D_{LCO} diffusion capacity for carbon monoxide, *FeNO* fractional exhaled nitric oxide, FEV_1 forced expiratory volume in one second, *FVC* forced vital capacity, *Ig* immunoglobulin, V_A alveolar volume

Table E2. Baseline characteristics of patients with induced sputum and those with spontaneous sputum samples

	**Induced sputum N = 31**	**Spontaneous sputum N = 81**	**P value**
Males, n (%)	20 (65)	68 (84)	0.04
Age, years	71.7 ± 8.2	72.1 ± 9.1	0.53
Body mass index, kg/m^2	23.6 ± 3.4	23.3 ± 3.6	0.96
Smoking: current/ex/never, n	3/16/12	10/54/17	0.16
Asthma/ACO/COPD, n	4/23/4	9/44/28	0.07
Number (%) of patients who had exacerbations† in the previous year†	6 (19)	22 (27)	0.47
$\%FEV_1$, %	71 ± 19	69 ± 18	0.67
Sputum eosinophil, % (n = 73)	13 ± 21	11 ± 15	0.91
Sputum neutrophil, % (n = 73)	69 ± 25	77 ± 21	0.27
Exhaled nitric oxide, ppb (n = 107)	51 ± 38	44 ± 41	0.25
Blood eosinophils, cells/µL	309 ± 209	349 ± 381	0.93
Blood neutrophils, cells/µL	3986 ± 1265	3885 ± 1097	0.75
Serum total IgE, IU/mL	1019 ± 1864	470 ± 1086	0.009
Regular ICS use, n (%)	29 (94)	57 (70)	0.01
ICS dose, µg/day‡	496 ± 315	300 ± 309	0.002
Relative abundance of			
Phylum Bacteroidetes, %	18.7 ± 8.9	22.2 ± 9.4	0.04
Phylum Proteobacteria, %	21.5 ± 14.4	18.4 ± 12.3	0.17
Phylum Firmicutes, %	30.8 ± 8.0	33.5 ± 9.3	0.22
Class Bacteroidia, %	17.5 ± 8.5	21.0 ± 9.3	0.04
Class γ Proteobacteria, %	8.4 ± 14.0	8.2 ± 10.5	0.71
Class Bacilli, %	18.4 ± 9.2	20.6 ± 11.9	0.59
Genus *Porphyromonas*, %	2.3 ± 2.2	4.2 ± 4.2	0.053
Genus *Haemophilus*, %	5.8 ± 13.2	6.3 ± 9.9	0.31
Genus *Strepotococcus*, %	15.0 ± 8.9	16.0 ± 8.3	0.55

Mean ± SD †Cases of exacerbations that required antibiotics, systemic corticosteroids, emergency room visits, or hospitalisation. ‡Equivalent to fluticasone propionate. ACO; asthma-COPD overlap, ICS; inhaled corticosteroid.

Table E3. Multivariable analysis for the diagnosis of ACO vs COPD

	Model 1			Model 2		
Variables	**Odds**	**95% CI**	**P value**	**Odds**	**95% CI**	**P value**
Age, year	0.89	0.80, 1.001	0.052	0.91	0.82, 1.02	0.12
Male sex	4.06	0.48, 34.2	0.20	2.61	0.32, 21.4	0.37
Pack-year	0.99	0.97, 1.01	0.44	0.99	0.97, 1.02	0.62
Serum total IgE, IU/mL	1.00	0.999, 1.003	0.30	1.00	0.999, 1.003	0.32
Relative abundance of *Porphyromonas*, %	0.78	0.65, 0.94	0.01	-	-	-
Relative abundance of *Fusobacterium*, %	-	-	-	0.73	0.57, 0.94	0.02
Daily dose of ICS, µg	1.01	1.003, 1.01	0.0004	1.01	1.004, 1.01	0.0001
Type of sputum sampling, spontaneous	0.71	0.14, 3.53	0.94	0.83	0.15, 4.76	0.84
			$R^2 = 0.47$			$R^2 = 0.46$

CI: confidence interval, ICS: inhaled corticosteroid, equivalent to fluticasone propionate.

Table E4. Multivariable analysis for eosinophil-low phenotype (sputum eosinophil < 2%) vs remaining (sputum eosinophil ≥ 2%)

	Odds	95%CI	P value
Relative abundance of Bacteroidia, %	1.07	1.001, 1.14	0.046
Type of sputum sampling, spontaneous	0.38	0.12, 1.20	0.10
Daily ICS doses, μg	0.999	0.997, 1.0004	0.14

CI: confidence interval, ICS: inhaled corticosteroid, equivalent to fluticasone propionate.

Table E5. Correlation coefficients between the relative abundance of bacteria and sputum cell counts or other indices

	Rho	P value	Rho	P value	Rho	P value
**Phylum**	**Bacteroidetes**		**Proteobacteria**		**Firmicutes**	
Exhaled nitric oxide, ppb	-0.20	0.04*	0.17	0.09	-0.08	0.44
Sputum neutrophils, %	0.11	0.35	0.13	0.27	-0.08	0.51
Sputum eosinophils, %	-0.23	0.055	0.13	0.26	-0.04	0.74
Sputum (neutrophils – eosinophils), %	0.15	0.21	0.08	0.48	-0.06	0.61
Blood eosinophils, cell/μL	-0.24	0.01*	0.15	0.12	0.10	0.31
%FEV_1, %	0.16	0.10	0.12	0.19	-0.22	0.02*
Exacerbation in the previous year†	-0.09	0.34	-0.08	0.41	0.15	0.12
**Class**	**Bacteroidia**		**γ Proteobacteria**		**Bacilli**	
Exhaled nitric oxide, ppb	-0.24	0.02*	-0.01	0.90	0.38	0.70
Sputum neutrophils, %	0.11	0.32	0.35	0.002*	-0.13	0.29
Sputum eosinophils, %	-0.24	0.04*	-0.06	0.64	0.21	0.07
Sputum (neutrophils – eosinophils), %	0.15	0.19	0.32	0.006*	-0.17	0.16
Blood eosinophils, cell /μL	-0.21	0.03*	0.07	0.49	0.27	0.004*
%FEV_1, %	0.13	0.18	0.02	0.81	-0.22	0.02*
Exacerbation in the previous year†	-0.07	0.45	-0.05	0.57	0.22	0.02*
**Genus**	***Porphyromonas***		***Haemophilus***		***Streptococcus***	
Exhaled nitric oxide, ppb	-0.22	0.02*	-0.05	0.63	0.05	0.62
Sputum neutrophils, %	0.06	0.61	0.33	0.005*	-0.14	0.25
Sputum eosinophils, %	-0.11	0.36	-0.09	0.44	0.19	0.10
Sputum (neutrophils – eosinophils), %	0.07	0.54	0.31	0.008*	-0.17	0.16
Blood eosinophils, cell /μL	-0.06	0.55	0.03	0.76	0.26	0.006*
%FEV_1, %	0.21	0.03*	0.12	0.21	-0.22	0.02*
Exacerbation in the previous year†	-0.03	0.75	-0.12	0.20	0.23	0.01*

* $p < 0.05$

† Exacerbations that required antibiotics

Table E6. Multivariable analysis for modestly elevated sputum eosinophil group (2%≤ to <8%) vs high eosinophil group (8%≤)

	**Odds**	**95%CI**	**P value**
Relative abundance of *Haemophilus*, %	1.24	1.01, 1.52	0.042
COPD, reference	-	-	-
Asthma	1.55	0.19, 12.7	0.36
ACO	0.45	0.09, 2.34	0.15

CI: confidence interval

Table E7. Baseline characteristics stratified by the degree of airflow limitation

	$\%FEV_1 \geq 50\%$ N = 91	$\%FEV_1 < 50\%$ N = 21	P value
Males, n (%)	70 (77)	18 (86)	0.56
Age, years	72 ± 8	71 ± 11	0.94
Body mass index, kg/m^2	23.4 ± 3.3	23.6 ± 4.4	0.93
Smoking: current/ex/never, n	12/55/24	1/15/5	0.49
Asthma/ACO/COPD, n	12/55/24	1/12/8	0.39
Frequency of exacerbation needing antibiotics in the previous year	0.11 ± 0.31	0.81 ± 1.54	0.005
Frequency of exacerbation needing systemic corticosteroids in the previous year	0.29 ± 1.01	1.24 ± 2.83	0.02
Frequency of exacerbation needing hospitalisation in the previous year	0.00 ± 0.00	0.10 ± 0.30	0.003
Number (%) of patients who had exacerbations in the previous year†	19 (21)	9 (43)	0.050
Sputum eosinophil, % (n = 73)	4 (0-85)	4 (0-44)	0.71
Sputum neutrophil, % (n = 73)	80 (14-99)	87 (27-99)	0.32
Exhaled nitric oxide, ppb (n = 107)	48 ± 36	38 ± 54	0.02
Blood eosinophils, cells/μL	345 ± 308	355 ± 551	0.23
Blood neutrophils, cells/μL	3798 ± 884	3529 ± 492	0.33
Serum C-reactive protein, mg/dL	0.20 ± 0.5	0.32 ± 0.86	0.42
Serum total IgE, IU/mL	205 (0-8600)	58 (9-9822)	0.32
ICS dose, μg/day‡	336 ± 303	433 ± 392	0.45

Mean ± SD, Median (range). †Cases of exacerbations that required antibiotics, systemic corticosteroids, emergency room visits, or hospitalisation. ‡Equivalent to fluticasone propionate. ACO; asthma-COPD overlap, ICS; inhaled corticosteroid.

Table E8. Multivariable analysis for severe airflow limitation (%FEV_1<50%) vs %$FEV_1 \geq$ 50%

	**Odds**	**95% CI**	**P value**	**Odds**	**95% CI**	**P value**
Relative abundance of *Streptococcus*, %	1.07	1.001, 1.14	0.034	-	-	-
Relative abundance of *Porphyromonas*, %	-	-	-	0.83	0.69, 0.99	0.041
COPD, reference	-	-	-	-	-	-
Asthma	0.13	0.01, 1.39	0.21	0.17	0.02, 1.59	0.22
ACO	0.29	0.08, 1.02	0.79	0.39	0.13, 1.21	0.95

CI: confidence interval

Table E9. Multivariable analysis for sputum symptoms (≥3 scores of sputum item of CAT questionnaire) vs no or few sputum symptoms (<3 scores)

Baseline factors	**Odds**	**95% CI**	**P value**
Relative abundance of *Haemophilus*, %	1.07	1.02, 1.14	0.012
Relative abundance of *Streptococcus*, %	1.06	0.996, 1.13	0.07
Sputum eosinophil, %	1.04	1.01, 1.08	0.016
			R^2 = 0.21

CAT: COPD assessment test, CI: confidence interval

Figure legends

Figure E1. Flow chart of patient recruitment. FVC, forced vital capacity.

Figure E2. β-diversity analysis at the genus level assessed by PERmutational Multivariate Analysis of Variance of weighted UniFrac distances. Asthma (blue plots), ACO (red plots), and COPD (green plots).

Figure E3. Sputum microbiota in patients with pauci-granulocytic, eosinophilic, mixed granulocytic, and neutrophilic subtypes. Relative abundances of bacteria at levels of A) Phylum, B) Class, and C) Genus. *: The relative abundances of class γ-Proteobacteria*, and genus *Haemophilus** differed significantly across the four groups ($p < 0.05$ by the Kruskal-Wallis test).

Figure E4. Sputum microbiota in patients with severe airflow limitation (%FEV$_1$ <50%) and the remaining (%FEV$_1$ ≥50%). Relative abundances of bacteria at levels of A) Phylum, B) Class, and C) Genus. *: The relative abundances of phylum Firmicutes*, class Bacilli*, and genera *Streptococcus**, *Porphyromonas***, and *Haemophilus**** differed significantly between the two groups ($p < 0.05$ by the Wilcoxon rank-sum test).

Figure E5. A) Frequency of patients who had at least one exacerbation necessitating antibiotics in the previous year in patients within the top quartile of *Streptococcus* abundance and the remaining. Two group comparison was made using the Wilcoxon rank-sum test. B) Blue bars indicate patients with asthma; red bars, ACO; green bars, COPD.

Figure E6. Associations between inhaled corticosteroid dose (equivalent to fluticasone propionate, μg/day) and the relative abundance of genus *Pseudomonas*, A) in patients treated with regular oral corticosteroid (n = 11; 2 asthma and 9 ACO) and B) those without oral corticosteroid treatment (n = 101).
