# Supplementary figures and images for: Sputum microbiota and inflammatory subtypes in asthma, COPD, and its overlap
Source: J Allergy Clin Immunol Glob. 2023 Nov 21;3(1):100194. doi: 10.1016/j.jacig.2023.100194 (PMC10753087; doi:10.1016/j.jacig.2023.100194)

Figure E1

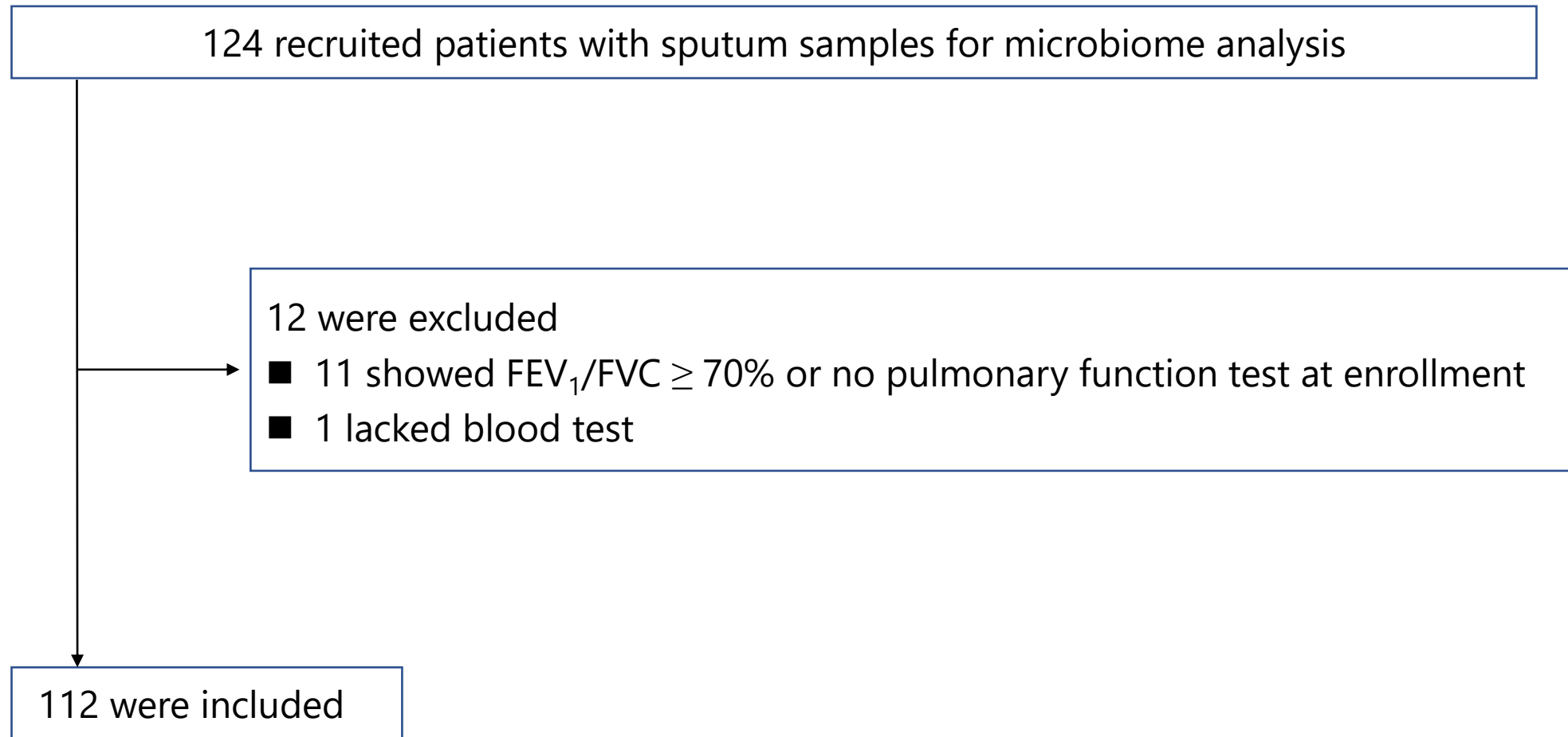

Figure E2

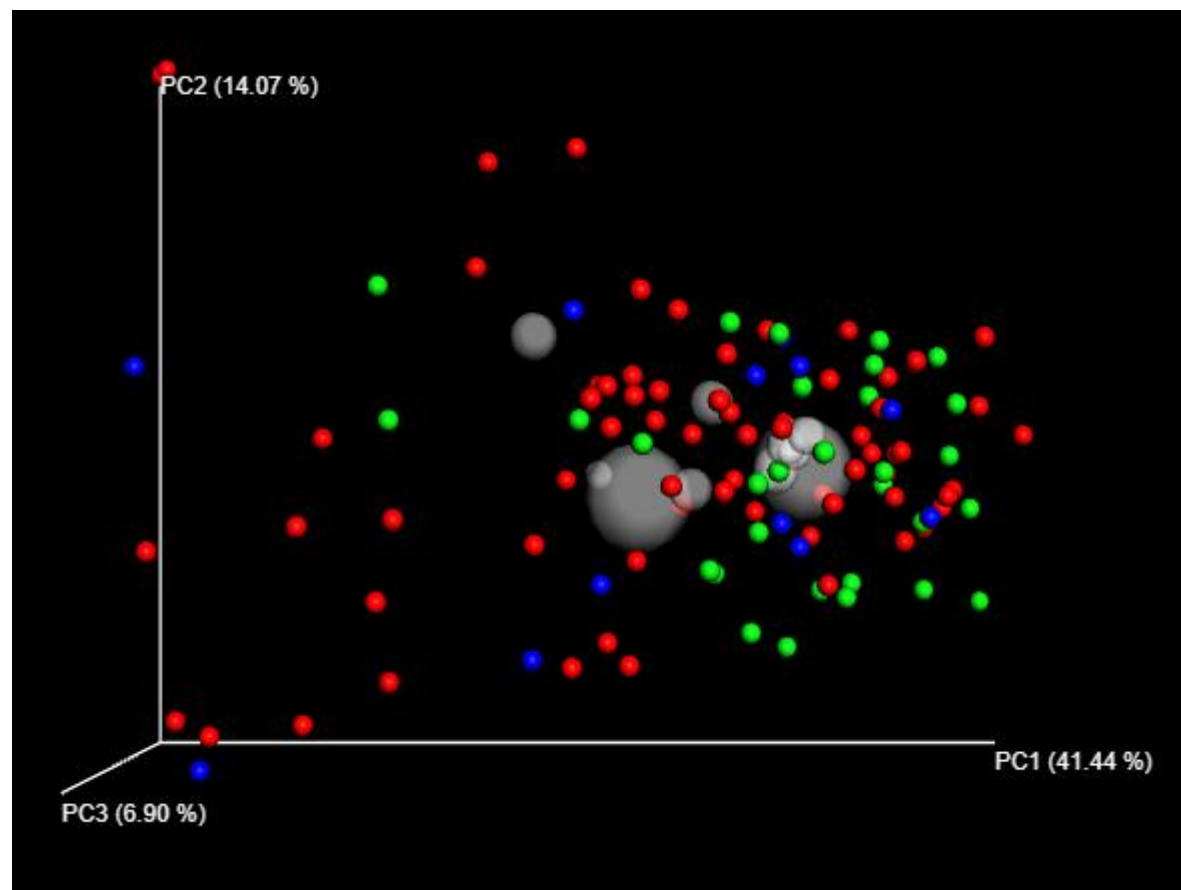

Figure E3

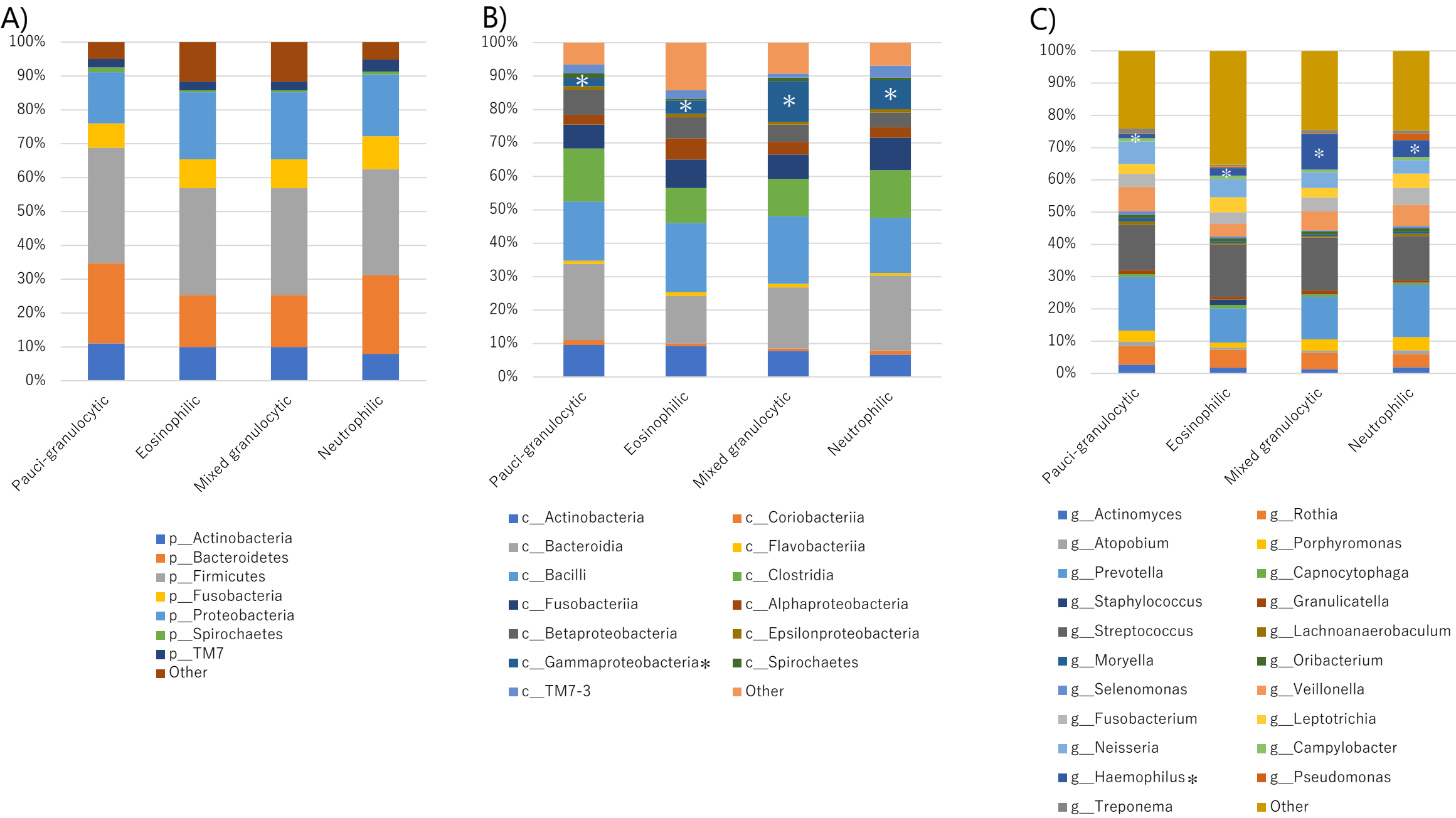

Figure E4

A)

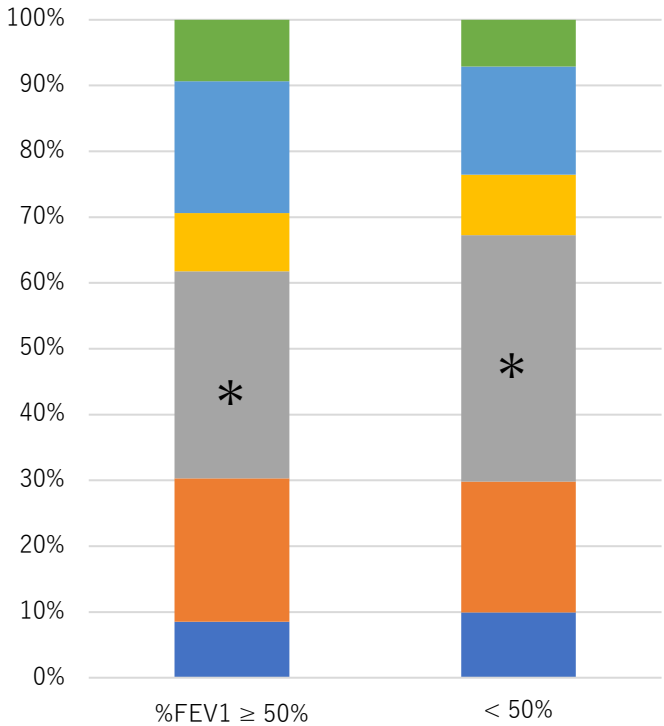

B)

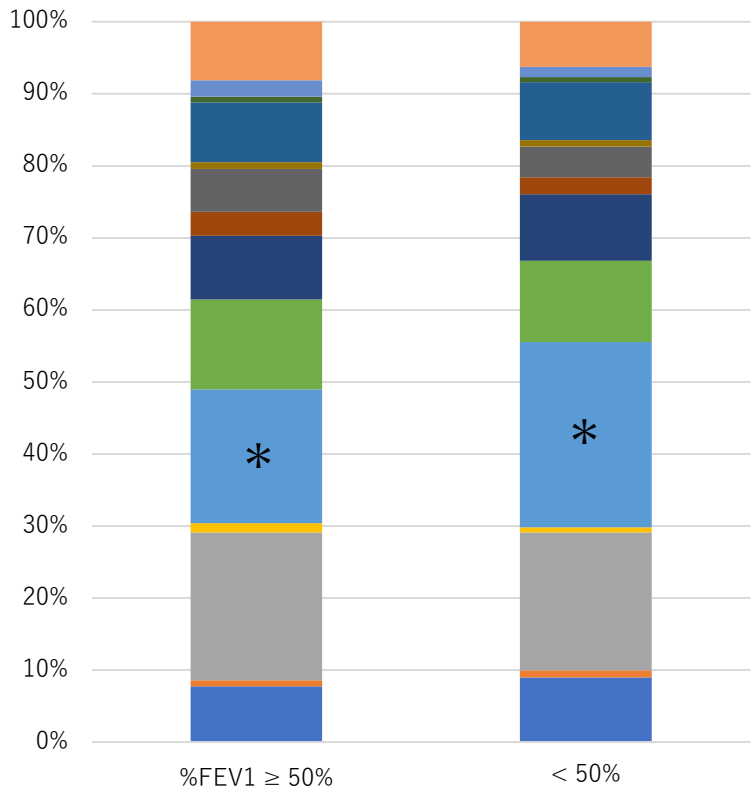

C)

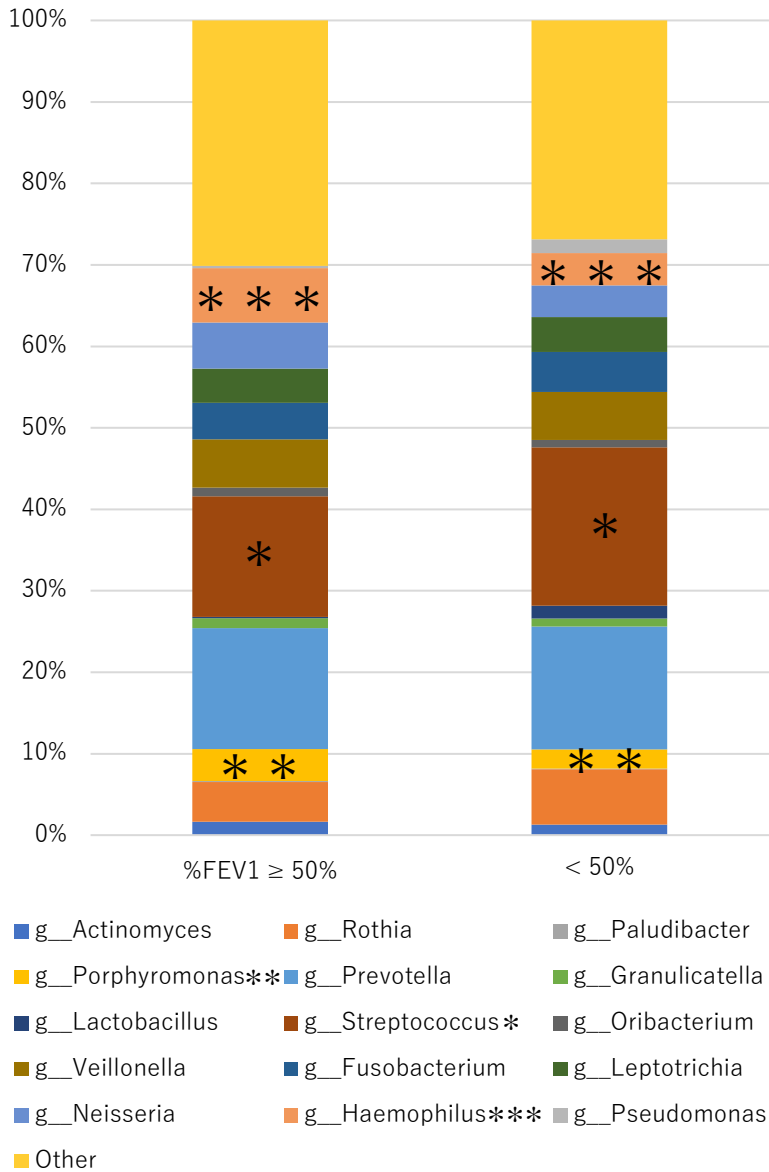

Figure E5

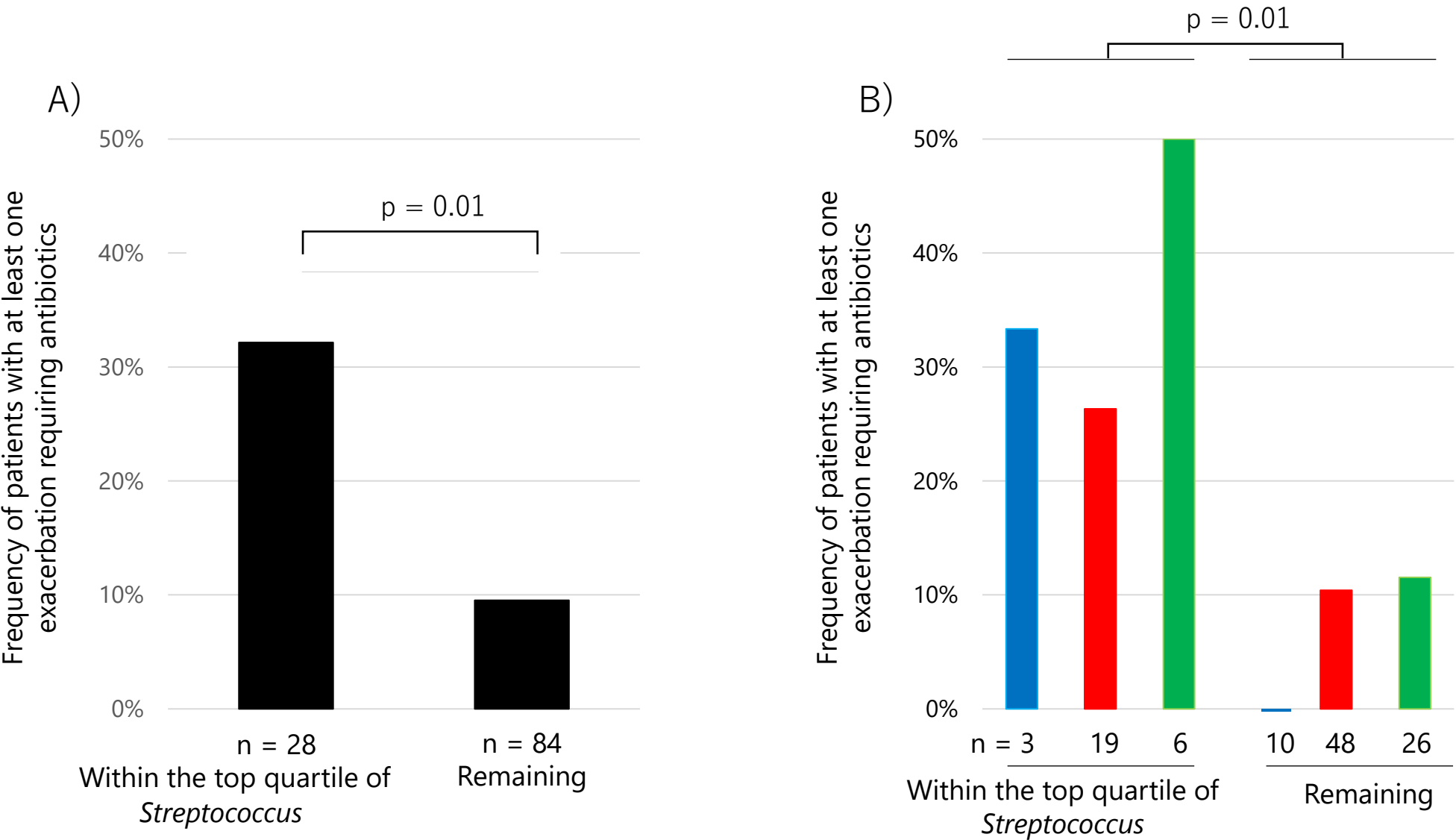

Figure E6

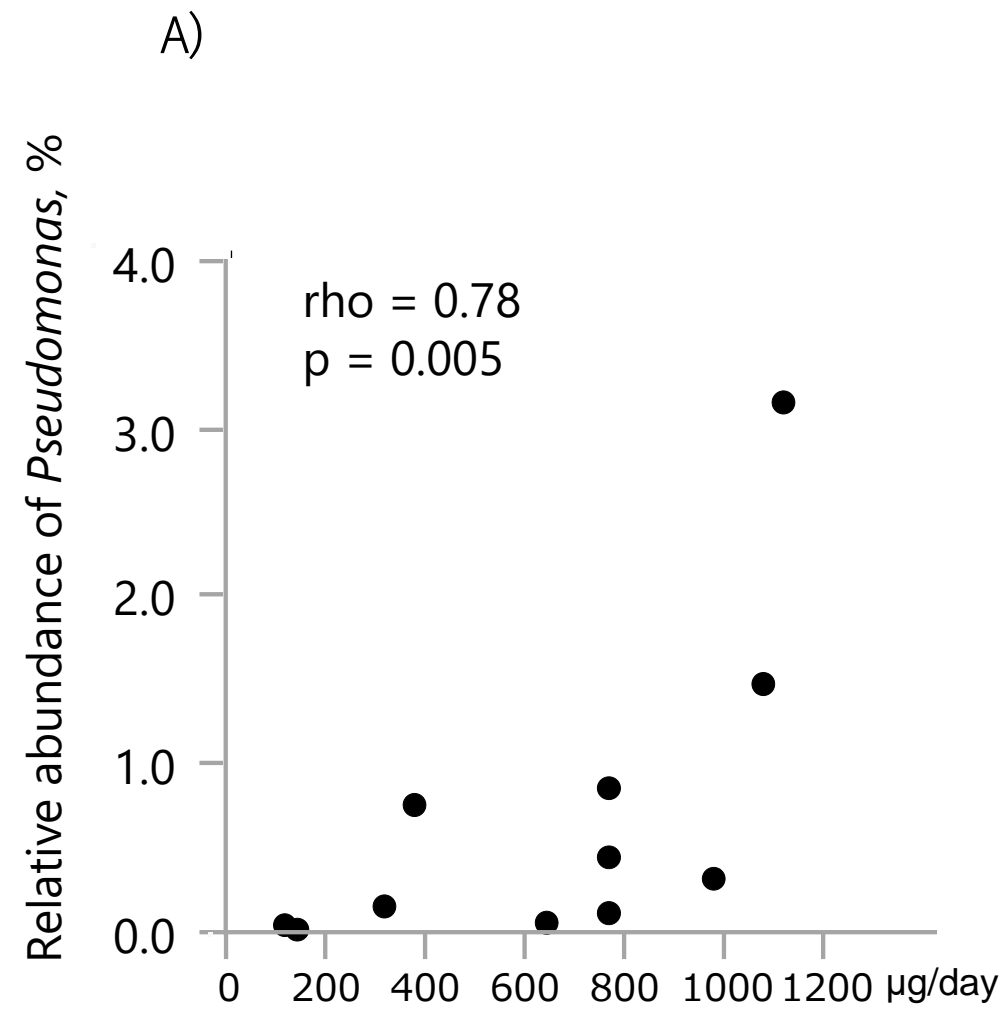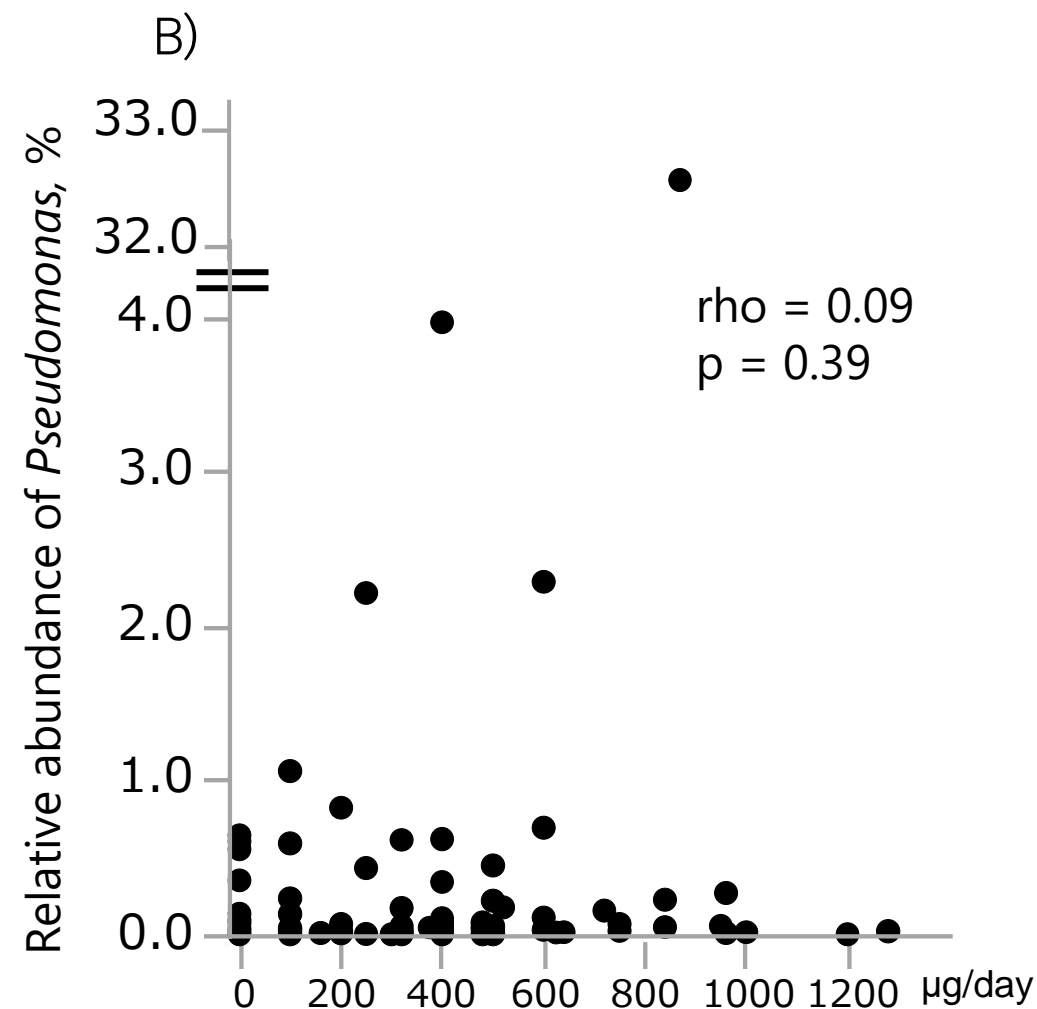

Supplement: Supplementary Figure [file mmc2.pdf]
